# Diagnosis of urinary tract infection in the emergency department – a real infection or an alarming indication for comprehensive geriatric assessment? A retrospective follow-up study

**DOI:** 10.1016/j.tjfa.2026.100208

**Published:** 2026-09-12

**Authors:** Laura Soikkeli, Marika Salminen, Tero Vahlberg, Laura Viikari, Ulla Hohenthal, Matti Viitanen, Maarit Wuorela

**Affiliations:** a Department of Geriatric Medicine, Faculty of Medicine/Clinical Medicine, University of Turku and Turku University Hospital, The Wellbeing Services County of Southwest Finland, Turku, Finland; b Academic Health and Social Services Centre, The Wellbeing Services County of Southwest Finland, Turku, Finland; c Faculty of Medicine/Clinical Medicine, Department of General Practice, University of Turku, Turku, Finland; d Faculty of Medicine, Department of Clinical Medicine, Unit of Biostatistics, University of Turku and Turku University Hospital, Turku, Finland; e Tyks Acute/Turku University Hospital, The Wellbeing Services County of Southwest Finland, Turku, Finland; f Department of Infectious Diseases, University of Turku and Turku University Hospital, Turku, Finland; g Division of Clinical Geriatrics, Center for Alzheimer Research, Department of Neurobiology, Care Sciences and Society, Karolinska Institutet, Stockholm, Sweden; h Department of Nephrology, University of Turku and Turku University Hospital, Turku, Finland

**Keywords:** Older adults, Urinary tract infection, Asymptomatic bacteriuria, Urinalysis, Emergency department

## Abstract

**Purpose:**

To investigate whether urinary tract infection (UTI) diagnoses assigned in the emergency department (ED) were true UTI infections, characterize hospitalized UTI patients, and analyze the 12-month mortality and institutionalization rates.

**Methods:**

A retrospective study among older adults (75+) who were admitted to the ED in 2017 and diagnosed UTI by the ED physician. Patients were categorized into four groups based on the level of UTI diagnosis and the follow-up treatment location: discharged cystitis, hospitalized cystitis, discharged pyelonephritis, and hospitalized pyelonephritis. Group differences were analyzed using Chi-squared, Fisher´s exact, or Mann-Whitney U tests. Logistic regression adjused for confounders was used to compare symptoms. Kaplan-Meier and Cox regression analyses were applied to asses mortality and institutionalization during the 12-month follow-up.

**Results:**

Of 658 ED-diagnosed UTIs, only 354 (54%) had significant bacterial growth in urine cultures. Nevertheless, antibiotics were prescribed in 98% of cases. Institutionalization was significantly higher among hospitalized (31%) than among discharged (11%) cystitis patients (log-rank test, p < 0.001); age, Carlson Comorbidity Index and frailty adjusted HR=2.72, 95% CI 1.62–4.55, p < 0.001). Interestingly, hospitalized cystitis patients had higher institutionalization rates (31%) than hospitalized pyelonephritis patients (15%) (log-rank test, p = 0.003; age and gender adjusted HR=0.52, 95% CI 0.31–0.87, p = 0.014).

**Conclusions:**

In ED UTIs are frequently overdiagnosed in older adults, often masking more complex underlying conditions. Because a hospitalization for cystitis serves as a major red flag for institutionalization within a year, these patients require an urgent comprehensive geriatric assessment rather than just a standard infection diagnosis.

## 1 Background

In older adults, both incidence of urinary tract infections (UTIs) and the resulting hospital admission rates are steadily rising [[1], [2], [3]]. Previous studies have shown that clinically diagnosed UTI and lower respiratory tract infections can predict disability in activities of daily living (ADL) in older adults [4]. UTI has also been associated with increased 30-day mortality [5]. Furthermore, among patients with diabetes, frailty has been linked to a higher risk of UTI development [6].

It is crucial to acknowledge that UTIs are often overdiagnosed and overtreated in older adults [[7], [8], [9], [10]]. For instance, the use of fluoroquinolone therapy has increased, with UTIs being one of the leading causes of unnecessary antibiotic prescriptions [11,12]. Fluoroquinolone exposure is a well-established modifiable risk factor for *Clostridium difficile* infection, which occurs more frequently and with greater severity in older adults [13,14]. In addition, unnecessary use promotes antimicrobial resistance among urinary tract pathogens, particularly in this population [[15], [16], [17]].

Diagnosis of UTI is based on the presence of clinical symptoms combined with significant bacteriuria [18], and UTIs are classified as cystitis or pyelonephritis depending on the level of infection [19]. Urine culture remains the standard diagnostic test, but results are not available during emergency department (ED) visits, necessitating empiric treatment. Currently, urine samples are often screened by flow cytometry before culturing to expedite diagnostics and reduce the need for urine cultures [20]. However, urine dipstick testing has been shown to be unreliable for diagnosing UTI in older adults [21].

It is important to note that UTI diagnosis cannot rely solely on urine culture results because of the high prevalence of asymptomatic bacteriuria (ASB) in the older adults. The prevalence of ASB varies widely, ranging from 4% to 19% in older men and from 11% to 16% in older women, and is most common among the frailest individuals. In long-term care settings, rates are even higher, ranging from 15% to 40% in men and from 25% to 40% in women [22].

Diagnosing symptomatic UTI in frail older adults is challenging, particularly in ED, where presentations are often nonspecific. Common findings include falls, delirium, malaise/lethargy, and bacteriuria without localized UTI symptoms [23,24]. Laboratory tests often fail to explain the declining general condition of these patients. Comprehensive assessment of medical history, medications, cognitive status is time-consuming and often constrained by the need for rapid decision-making and patient flow in the ED. Given the high prevalence of asymptomatic bacteriuria, UTI may be readily used as an explanatory diagnosis [25,26]. However, studies indicate that nonspecific symptoms do not substantially increase the likelihood of bacterial infection in older patients visiting the ED [27]. Moreover, there is insufficient evidence that antimicrobial treatment improves outcomes such as delirium in older patients [28].

The aims of this study were to investigate: 1) whether UTI diagnoses assigned in the ED were true UTI infections, 2) the characteristics of hospitalized UTI cases, and 3) the 12-month mortality and institutionalization rates among UTI patients diagnosed in the ED. Institutionalization rates due to infections other than UTIs were also investigated.

## 2 Methods

### 2.1 Study design and population

**Fig. 1:**
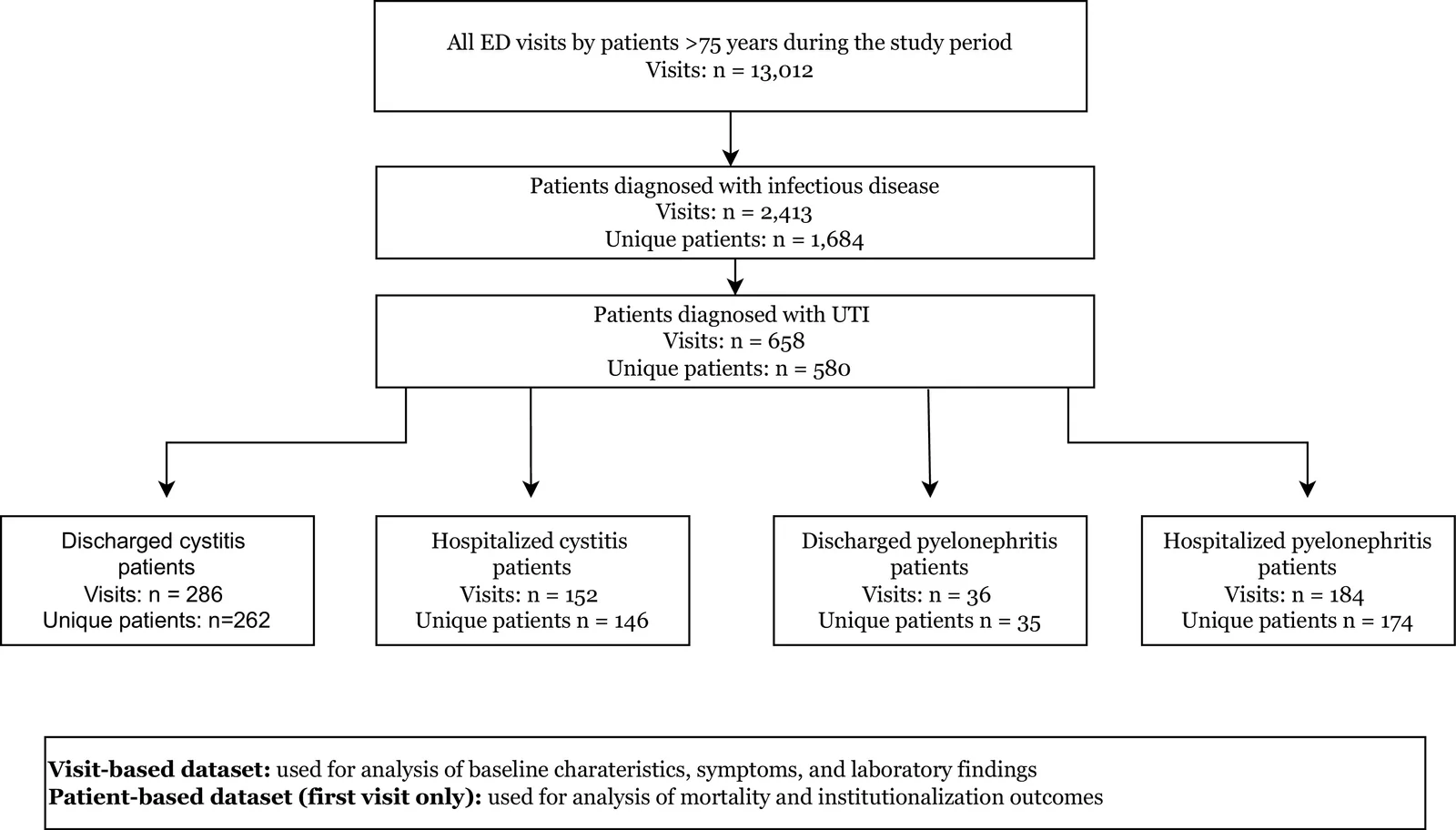
Flowchart of study participants.

This was a retrospective follow-up study conducted among older citizens (aged 75+) of the city of Turku (population 188,636) in Finland who were admitted to the ED of Turku University Hospital during 2017 (n = 13,012). Of those, 2413 (19%) were diagnosed with an infectious disease by the ED physician. These visits were selected from the Turku University Hospital database by Auria Clinical Informatics, Turku University Hospital. Among these visits, 658 (27%) visits (in 580 patients) had UTI as the primary diagnosis. Patients were selected solely based on the discharge diagnosis recorded in the ED. The study is based on real-life data and no stricter diagnostic criteria were applied for patient selection, and no exclusion criteria were used (Fig. 1).

To investigate mortality and institutionalization, we used the number of patients. A total of 1684 patients were diagnosed with an infection. Patients with UTI (n = 580) were selected and followed up for mortality and institutionalization over 12 months. Patients with infections other than UTI (n = 1104) were followed up for institutionalization over the same period.

The study data consisted of ED visits and were analyzed on two levels: visit-level and patient-level (Fig. 1). Visit-level data were used to analyze clinical presentations, laboratory results, and baseline demographic information such as age, frailty and CCI. In these analyses, each ED visit was treated as a separate observation, regardless of whether the same patient had multiple visits during the study period. Patient-level analyses were conducted for outcomes such as mortality and admission to a nursing home. For these analyses, only the patient’s first ED visit during the study period was included to avoid bias due to multiple visits by the same individual. Thus, each patient contributed only one observation to the patient-level analyses.

### 2.2 Infection diagnoses assigned in the ED

#### 2.2.1 Urinary tract infection

UTI diagnoses assigned in the ED were identified using ICD-10 codes. UTI cases were categorized by diagnosis level into (1) pyelonephritis (N10, N12) and (2) cystitis (N30.0, N30.8, N30.9, N39.0) and by follow-up treatment location (1) discharged and (2) hospitalized. Accordingly, four patient groups were formed: 1. discharged cystitis, 2. hospitalized cystitis, 3. discharged pyelonephritis, and 4. hospitalized pyelonephritis patients.

#### 2.2.2 Other infections

All infections diagnosed by the ED physician were divided into six groups as follows: 1) UTIs 2) skin infections (ICD 10 codes A46, B00, B01, B02), 3) sepsis (ICD codes A40-41), 4) unspecified bacterial infection (ICD code A49), 5) gastrointestinal tract infections (ICD codes A00-09, K81, K80, K57.2, K57.3), and 6) respiratory tract infections (ICD codes J00-J06, J09-J18, J20-J22). Infections were diagnosed according to the national treatment guidelines [[29], [30], [31]].

### 2.3 Symptoms and measurements

Symptoms (increased urinary frequency, urinary urgency, dysuria, delirium, fever [over 37.5 °C], falls, malaise, back pain, nausea, vomiting, stomach ache) were extracted from the patient´s medical records by reviewing the admission notes. Laboratory results for creatinine-based glomerular filtration rate (GFR), leukocytes, C-reactive protein (CRP), and urine dipstick tests were extracted from the Turku University Hospital database by Auria Clinical Informatics, Turku University Hospital.

Urine samples were obtained either through spontaneous voiding or by catheterization. All urine samples were initially analyzed using flow cytometry-based analyzers to detect bacteria and leukocytes [20]. In women, screening was considered positive if the white blood cell count was ≥11 cells/μl or if there were ≥200 cells/μl of bacteria. In men, the cutoff values were ≥37 cells/μl for white blood cells and ≥75 cells/μl for bacteria. All samples that tested positive in the screening test were cultured. A urine culture was considered to show significant bacterial growth when bacterial counts exceeded 10^5 CFU/mL. In patients presenting with symptoms of urinary tract infection, bacterial growth >10^4 CFU/mL was also regarded as significant. A urinary dipstick test was considered positive if it showed a positive nitrite result, leukocyte esterase, or both.

Data on age, gender, diabetes (ICD-10 codes E10–E14), dementia (ICD-10 codes F00–F03), and antibiotic treatment were also extracted from the Turku University Hospital database. Frailty and comorbidity data were gathered from the medical records of Turku City Hospital. Frailty was defined as a score of five or more on the Clinical Frailty Scale (CFS) [32,33](indicating dependence on another person for daily activities). Additionally, if a patient required home care for basic activities of daily living, they were considered frail. This classification was assigned retrospectively during data analysis. Comorbidity status was determined using the Charlson Comorbidity Index (CCI) [34,35]. Data on participants' diagnoses for CCI were gathered from the medical records of Turku City Hospital and Turku University Hospital from the last five years (between 1 January 2013 and 31 December 2017).

### 2.4 Mortality

Data from all participants who died before 1 January 2019 were obtained from the Finnish Cause of Death Registry using unique personal identification number.

### 2.5 Institutionalization

Institutionalization was defined as permanent entry into a long-term care facility before January 2019. Data on this were gathered from the municipality's electronic patient record system and coded by the month and year of entry.

### 2.6 Ethics

The study was conducted according to the guidelines of the Declaration of Helsinki. The Ethics Committee of the Hospital District of Southwest Finland approved the study protocol (diary number: 71/118,011/2017).

### 2.7 Statistical analysis

Differences between the groups were tested using Chi-squared test and Fisher´s Exact test in categorical variables and the Mann-Whitney U Test in continuous variables. Logistic regression was performed to compare the most common symptoms (increased urinary frequency/urgency/dysuria, delirium and fever) between cystitis patients who were discharged and those hospitalized after adjusting for potential confounders (age, CCI, frailty). Leukocytes and CRP values were compared using analysis of covariance (ANCOVA) after adjustment for potential confounders (age, CCI, frailty). Log-transformed values for leukocytes and CRP were used in ANCOVA due to positively skewed distributions.

The Kaplan-Meier method was employed to estimate mortality and institutionalization during the 12-month follow-up. Log-rank tests were used to compare mortality and institutionalization curves between groups (discharged vs. hospitalized cystitis and hospitalized cystitis vs. hospitalized pyelonephritis). Cox regression analysis was used to test differences in mortality and institutionalization between the groups after adjusting for potential confounders. P-values < 0.05 were considered statistically significant. All statistical analyses were performed using SAS System for Windows, version 9.4 (SAS Institute Inc., Cary, NC, USA).

## 3 Results

### 3.1 Baseline characteristics of the UTI patients

During the year 2017, 580 older adults were admitted to the ED and diagnosed with UTI a total of 658 times (UTI cases). The median age of the patients was 84.0 years (range 75–104 years). The majority (77%) were female. More detailed baseline characteristics of the 580 patients are shown in Appendix I.

### 3.2 Baseline characteristic of the UTI cases divided by the level of UTI and the follow-up treatment location

Hospitalized patients with cystitis (n = 152) were older, more often frail, and more likely to be multimorbid compared to those who were discharged (n = 286). The prevalence of dementia, diabetes, and kidney failure was higher among hospitalized cystitis cases than among discharged ones. However, no statistically significant differences in baseline characteristics were observed between the patients who were discharged and those who were hospitalized with pyelonephritis (Table 1). Patients hospitalized for cystitis were older, and more often females than patients hospitalized for pyelonephritis (Appendix II).

**Table 1:** Baseline characteristic of the UTI^a^ cases divided by the level of UTI and the follow-up treatment location (n = 658).

	Cystitis		p-value^b^	Pyelonephritis		p-value
	Discharged (n = 286) n (%)	Hospitalized (n = 152) n (%)		Discharged (n = 36) n (%)	Hospitalized (n = 184) n (%)	
Age, Md (IQR), range	84.0 (80.0–88.0), 75–100	86.0, (81.0–91.0), 75–104	<0.001	82.0 (78.5–86.0), 75–92	83.0 (79.0–88.0), 75–97	0.132
Female	227 (79)	127 (84)	0.290	29 (81)	119 (65)	0.063
Charlson Comorbidity Index >3	128 (45)	104 (68)	<0.001	22 (61)	121 (85)	0.593
Frailty^c^ (n = 652)	141 (49)	91 (61)	0.021	19 (53)	106 (58)	0.545
Diabetes^d^	44 (15)	36 (24)	0.032	8 (22)	54 (29)	0.385
Dementia^e^	67 (23)	56 (37)	0.003	13 (36)	55 (30)	0.460
GFR^f^ (n = 500)			0.041			0.081
GFR >60	58 (38)	43 (29)		3 (14)	63 (35)	
GFR 60–45	54 (36)	47 (32)		9 (41)	43 (24)	
GFR <45	39 (26)	58 (39)		10 (46)	73 (41)	

a Urinary tract infection.

b Differences between the groups were tested using the Chi-squared test or Fisher's exact test for categorical variables and the Mann-Whitney U test for the continuous variable.

c Clinical Frailty Scale ≥5.

d ICD-10 codes E10-E14.

e ICD-10 codes F00-F03.

f Glomerular filtration rate Md = median IQR = interquartile range.

### 3.3 Symptoms and laboratory findings of the UTI cases divided by the level of UTI and the follow-up treatment location

Urinary tract symptoms were statistically significantly more common among the discharged patients than among those who were hospitalized for cystitis, whereas delirium, falls, malaise, back pain, nausea, and stomachache were statistically more frequent among hospitalized cystitis patients compared to those who were discharged (Table 2). Additionally, blood levels of leukocytes and CRP were higher in hospitalized patients with cystitis who were hospitalized compared to those who were discharged. After adjusting for age, CCI, and frailty, differences in urinary tract symptoms, delirium, fever, leukocytes, and CRP between patients who were discharged and those who were hospitalized for cystitis remained significant (Table 3).

**Table 2:** Differences in symptoms and laboratory findings among UTI^a^ cases divided by the level of UTI and the follow-up treatment location (n = 658).

	Cystitis		p-value^b^	Pyelonephritis		p-value^b^
	Discharged (n = 286) n (%)	Hospitalized (n = 152) n (%)		Discharged (n = 36) n (%)	Hospitalized (n = 184) n (%)	
Symptoms reported						
increased urinary frequency/urgency/dysuria	157 (55)	28 (18)	<0.001	14 (39)	37 (20)	0.029
Delirium	35 (12)	40 (26)	<0.001	5 (14)	23 (12.5)	0.787
fever (over 37.5 °C)	25 (9)	21 (14)	0.099	25 (69)	123 (67)	0.848
Fall	6 (2)	44 (29)	<0.001	2 (6)	41 (22)	0.021
Malaise	5 (2)	48 (32)	<0.001	3 (8)	56 (30)	0.007
Backpain	1 (0)	7 (5)	0.003	4 (11)	16 (9)	0.750
Nausea	1 (0)	11 (7)	<0.001	0 (0)	15 (8)	0.139
Vomiting	1 (0)	13 (9)	<0.001	5 (14)	18 (10)	0.549
stomach ache	4 (1)	10 (7)	0.007	7 (20)	18 (10)	0.089
Leukocytes, Md (IQR), range (n = 497)	8.0 (6.3–9.8), 1.6–19.4	8.8 (6.9–12.2), 1.9–22.2	<0.001	9.9 (9.2–13.4), 5.7–19.3	11.7 (9.4–16.1), 2.9–29.0	0.116
CRP^3^, Md (IQR), range (n = 499)	6.0 (2.0–20.0), 1.0–126.0	10.0 (3.0–27.5), 1.0–145.0	0.014	44.5 (34.0–91.0), 1.0–193.0	79.5 (37.0–136.0), 1.0–288.0	0.085
Urinary dipstick test, n (%) (n = 491)			0.415			0.292
Positive^d^	155 (93)	129 (96)		20 (100)	161 (95)	
Negative	11 (7)	6 (4)		0 (0)	9 (5)	
Culture, n (%) (n = 608)			0.422			0.734
Positive	139 (54)	80 (57)		23 (70)	112 (63)	
Negative/contaminated	119 (46)	60 (43)		10 (30)	65 (37)	

^a^Urinary tract infection.

^b^Differences between the groups were tested with Chi-squared test or Fisher´s Exact test in categorical variables and with Mann-Whitney U Test in continuous variables.

^c^C-reactive protein.

^d^positive nitrite result, leukocyte esterase, or both.

Md = median.

IQR = interquartile range.

**Table 3:** Adjusted differences in symptoms and laboratory findings among UTI^a^ cases divided by the level of UTI^a^ and the follow-up treatment location.

		Discharged cystitis vs. hospitalized cystitis (n = 438)	
	n	Adjusted^b^ OR (95%CI)	p-value
Symptoms reported			
increased urinary frequency /urgency/dysuria	434	4.15 (2.52–6.80)	<0.001
Delirium	434	0.41 (0.24–0.70)	0.001
fever (over 37.5 °C)	434	0.48 (0.25–0.95)	0.035
	n	Adjusted^b^ geometric mean (95% CI)	p-value
CRP^c^	292	6.36 (5.06–8.00) vs. 9.22 (7.31–11.64)	0.026
Leukocytes^c^	291	7.70 (7.23–8,20) vs. 9.15 (8.58–9.75)	<0.001

a Urinary tract infection.

b Adjusted for age, CCI, and frailty.

c Values are log-transformed OR = odds ratio CI = confidence interval.

Regarding pyelonephritis cases, falls and malaise were more common symptoms among hospitalized patients than among those who were discharged, but discharged patients had more UTI symptoms. Notably, both discharged and hospitalized pyelonephritis cases exhibited a median CRP level of over 40 mg/ml (Table 2).

The dipstick test was highly positive (93–100%) in all patients diagnosed with a UTI. A total of 518 samples were screened by flow cytometry, of which 485 (94%) tested positive in the screening assay. Of the 658 ED-diagnosed UTIs, only 354 (54%) had significant bacterial growth in their urine samples. In almost all cases (n = 648 [98%]), antibiotics were administered or prescribed in the ED.

### 3.4 Rates of mortality and institutionalization by UTI

**Fig. 2:**
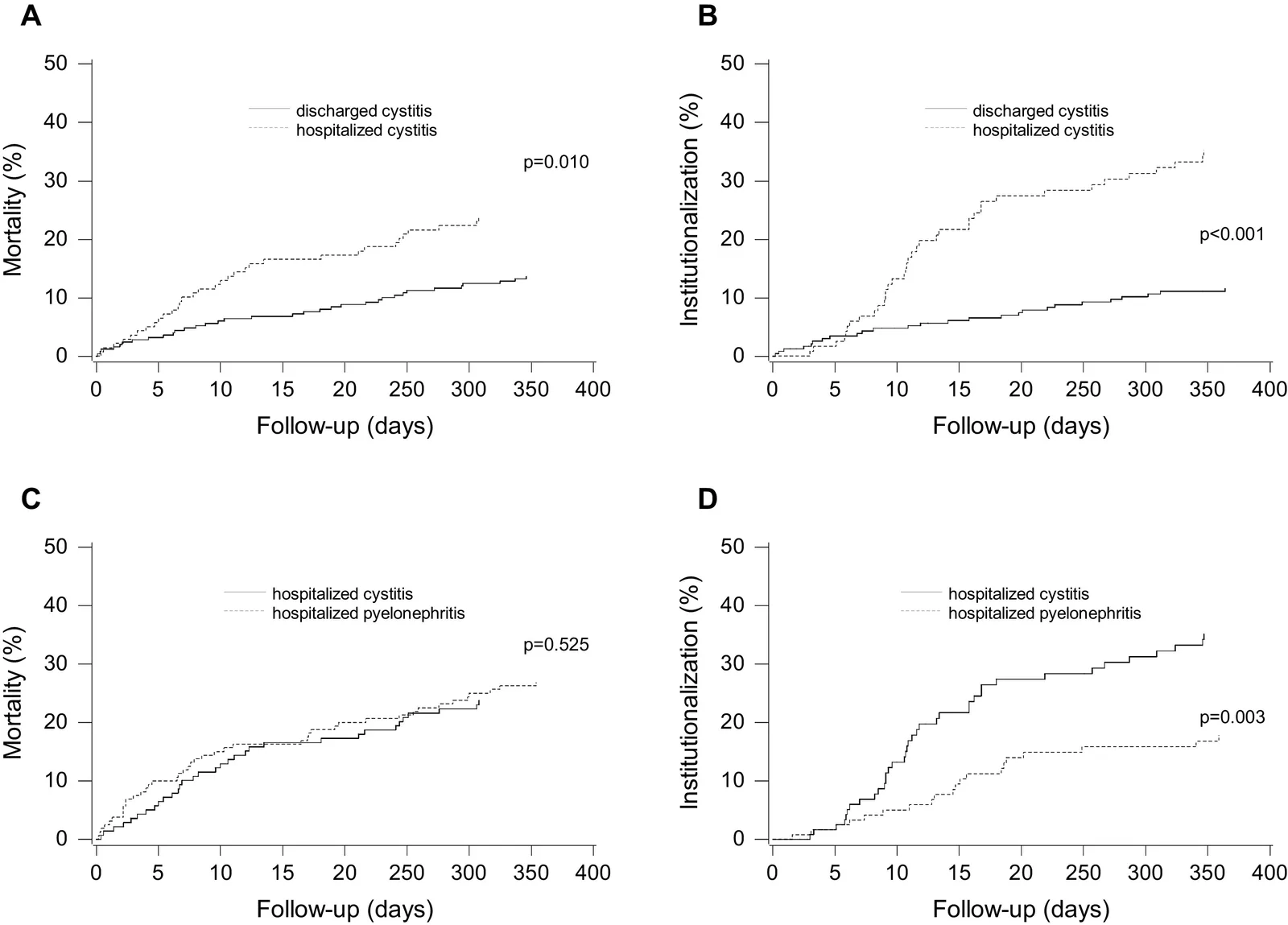
Kapplan meier estimates of mortality and institutionalization among UTI patients (n = 580) during the 12-month follow-up. Adjusted hazard ratios (HR), (95% confidence intervals [CI]) and p-values: A. Age, CCI and frailty -adjusted HR 1.26 (95% CI 0.77–2.07), p = 0.356. B. Age, CCI and frailty -adjusted HR 2.72 (95% CI 1.62–4.55), p < 0.001. C. Age and gender -adjusted HR 1.13 (95% Cl 0.71–1.81), p = 0.608. D. Age and gender -adjusted HR 0.52 (95% CI 0.31–0.87), p = 0.014.

Fig. 2. shows the rates of mortality and institutionalization by UTI during the 12-month follow-up. Twenty-four percent of hospitalized and 14% of discharged cystitis patients died during the follow-up (log-rank test, p = 0.010). The difference did not remain significant after adjustment for age, CCI, and frailty (HR = 1.26, 95% CI 0.77–2.07, p = 0.356). There was no significant difference in mortality between hospitalized cystitis and hospitalized pyelonephritis patients (log-rank test, p = 0.525; age- and gender-adjusted HR = 1.13, 95% CI 0.71–0.87, p = 0.608).

Institutionalization was significantly higher among hospitalized (31%) than among discharged (11%) cystitis patients (log-rank test, p < 0.001; age, CCI, and frailty adjusted HR = 2.72, 95% CI 1.62–4.55, p < 0.001). Additionally, institutionalization of hospitalized pyelonephritis patients (15%) was significantly lower than that of hospitalized cystitis patients (31%) (log-rank test, p = 0.003; age- and gender-adjusted HR = 0.52, 95% CI 0.31–0.87, p = 0.014).

### 3.5 Rates of institutionalization by the type of infection

Overall, 16% of UTI patients were institutionalized during the 12-month follow-up period. Corresponding rates among patients with respiratory and skin infections were 6% and 8%, respectively. The rates of institutionalization by type of infection are shown in Appendix III.

## 4 Discussion

In our study population, less than half of the patients diagnosed with UTI showed no significant bacterial growth in urine cultures. Similar findings have been demonstrated in other studies as well. Due to diagnostic pitfalls, acute cystitis, in particular, is highly overdiagnosed and overtreated among older adults [[7], [8], [9], [10]]. One hundred and eighty-four patients diagnosed with pyelonephritis and admitted to the hospital were also included in our study. Hospitalized pyelonephritis patients exhibited high CRP counts (median 79.5 mg/L) and fever (67%), suggesting some other bacterial infection requiring antibiotic treatment, despite negative urine cultures in 37% of the cases. Nevertheless, these infections should be specified for more accurate treatment.

Urine dipstick tests, which were previously commonly used for diagnosis of UTI in the ED, have proven to be unreliable in older adults [21]. In our study, urine samples were additionally screened for leukocytes, red blood cells, or bacteria using flow cytometry. Contrary to a previous study, this supplementary screening did not appear to improve diagnostic accuracy. Consequently, our results raise the question of how reliable urine sample screening with flow cytometry is in older adults, and this requires further investigation [20].

Antibiotic treatment was administered to almost all patients (648 out of 658; 98%) based on positive screening or dipstick results. Such liberal antibiotic use may increase the risk of adverse outcomes, including colonization and infection with healthcare-associated pathogens such as methicillin-resistant Staphylococcus aureus (MRSA) and Clostridium difficile [36,37]. Given the high prevalence of ASB in older adults, positive urine tests do not necessarily indicate infection requiring treatment. Evidence shows that treating ASB does not decrease the likelihood of future UTIs or reduce mortality rates but is associated with adverse drug effects, drug interactions, and antimicrobial resistance [[38], [39], [40], [41]]. For instance, trimethoprim use has been linked to acute kidney injury and hyperkalemia with potential cardiac complications [42]. The treatment of ASB is also associated with a longer duration of hospitalization after urine testing (10). Among hospitalized patients, delaying antibiotic treatment for cystitis until culture results are available and clinical assessment is completed may help reduce unnecessary antibiotic use and associated harms.

The frequent presence of ASB higlights the importance of recognizing the actual symptoms of disease. Although several diagnostic algorithms have been developed to enhance UTI diagnostic accuracy, their practical application has proven challenging. Most algorithms require either dysuria alone or fever for UTI diagnosis [43]. Previous studies have indicated dysuria as the most reliable symptom of UTI in the older adults with multiple comorbidities [44,45]. In our research, only 18% of hospitalized cystitis patients presented with lower urinary tract symptoms (urgency, dysuria, frequency). There is a widespread misconception that non-specific and non-localizing signs are clinical presentations of symptomatic UTI among the older adults [18]. In our study, a large proportion of patients also presented with non-specific symptoms such as malaise (32%), falls (29%), and changes in mental status (26%). These non-specific signs and symptoms could originate from various other health issues besides UTI.

In frail older patients, UTI may sometimes be used as a convenient diagnosis, potentially replacing a more comprehensive evaluation and increasing the risk of overlooking serious underlying conditions. Conversely, even after thorough hospital assessment, the primary reason for emergency admission may be unmet care needs requiring 24/7 nursing home care. As Finland’s elderly care policy aims to support independent living, identifying patients at high risk of institutionalization is essential. Our study found a high risk of institutionalization and death among patients hospitalized with cystitis. Compared with patients hospitalized for other infections, these patients were significantly more likely to enter a nursing home within one year. Thus, hospitalization for cystitis may sometimes indicate the need for a comprehensive geriatric assessment (CGA) rather than infection alone, as CGA has been shown to improve the likelihood of older hospitalized adults remaining at home [46]. We found a lower risk of institutionalization among patients hospitalized with pyelonephritis than among those hospitalized with cystitis. Patients with acute pyelonephritis may have greater physiological and immunological reserve, resulting in a more typical inflammatory response and clinically recognizable infection. In contrast, patients hospitalized with cystitis may represent a frailer population, in whom non-specific symptoms such as falls, delirium, or functional decline more often lead to hospitalization. Thus, the observed association may partly reflect differences in frailty and physiological resilience rather than the severity or reversibility of the infection itself.

Cystitis, as an infection, should not lead to such high mortality rates, raising concerns about the possibility of overlooking a more serious underlying illness. Our findings underscore the importance of critically re-evaluating UTI diagnoses in hospitalized patients, as the underlying cause for admission may have been misattributed and could involve conditions other than cystitis. It is crucial to investigate potential factors contributing to the frequent misdiagnosis of UTIs. Additionally, it is important to explore the feasibility of developing a new algorithm that could enhance the accuracy of UTI diagnosis, especially for older, frail patients presenting to the ED with nonspecific symptoms. It is also crucial to collect urine samples from older patients judiciously and only when clear indications are present. A positive sample can easily lead the diagnostic process in the wrong direction.

This study has several limitations. The diagnosis of UTI was made in a clinical setting by several different physicians, and therefore the study does not follow a strictly controlled research design. On the other hand, a key strength of this patient selection is that it is based in real-world conditions, as they occur in the fast-paced environment of an ED. This setting allows for easier identification of development areas as they emerge in actual clinical practice. Due to its retrospective design, our study relies on existing data collected for other clinical purposes. Data on patients' living arrangements and the availability of informal or family caregivers were unavailable, although these factors may influence decisions regarding permanent institutionalization and represent unmeasured confounders. The use of spontaneously voided urine samples may have increased the risk of contamination, potentially affecting the accuracy of microbiological findings. As an observational study, our findings demonstrate associations but cannot establish causality. Finally, the single-center design, relatively small sample size, and lack of racial diversity (all patients were Caucasian) may limit the generalizability of the results. Although the sample size was small, the study´s strength lies in the careful rewiew of medical records and comprehensive collection of patient data, including diseases, laboratory values and frailty status.

## 5 Conclusions

Our result show that older patients hospitalized with a diagnosis of cystitis have an increased risk of institutionalization within the following year. Hospitalization attributed to cystitis should not be viewed solely as an infectious episode but rather as a sentinel event signaling increased vulnerability. UTI is an indicator for CGA to address any unmet health and social care needs, maintain a good functional status and prevent progression of frailty.

## 6 Declarations

### Funding

This work was financially supported by ERVA funding from the City of Turku/Welfare Division, the Betania Foundation, the Uulo Arhio Foundation, and the Turku University Foundation. The financial sponsors played no role in the design, execution, analysis, or interpretation of the data.

### Ethics approval

The study was conducted according to the guidelines of the Declaration of Helsinki. The Ethics Committee of the Hospital District of Southwest Finland approved the study protocol (diary number: 71/1801/2017).

### Consent of publication

Not applicable.

### Availability of data and materials

The datasets used and/or analyzed during the current study are available from the corresponding author on reasonable request.

## Declaration of generative AI and AI-assisted technologies in the manuscript preparation process

During the preparation of this work the author used Microsoft Copilot in order to check grammar, spelling and improve the language and readability of the text. After using this tool, the author reviewed and edited the content as needed and takes full responsibility for the content of the published article.

Supplementary Information

Below is the link to the electronic supplementary material.

Appendix I. Baseline characteristics of the participants (n = 580)

Appendix II. Baseline differences between ED-diagnosed hospitalized cystitis cases and hospitalized pyelonephritis cases

Appendix III. Institutionalization rates (%) by type of infection during the 12-month follow-up (n = 1684)

## CRediT authorship contribution statement

**Laura Soikkeli:** Writing – original draft. **Marika Salminen:** Writing – review & editing, Writing – original draft. **Tero Vahlberg:** Formal analysis, Data curation. **Laura Viikari:** Writing – review & editing. **Ulla Hohenthal:** Writing – review & editing. **Matti Viitanen:** Writing – review & editing. **Maarit Wuorela:** Writing – review & editing, Supervision.

## Declaration of competing interest

All authors declare that they have no competing interests.
