# Supplementary material for: Diagnosis of urinary tract infection in the emergency department – a real infection or an alarming indication for comprehensive geriatric assessment? A retrospective follow-up study
Source: J Frailty Aging. 2026 Sep 12;15(5):100208. doi: 10.1016/j.tjfa.2026.100208 (PMC13587059; doi:10.1016/j.tjfa.2026.100208)
Supplement: Supplementary file 1 [file mmc1.zip › Appendix III in PDF format.pdf]

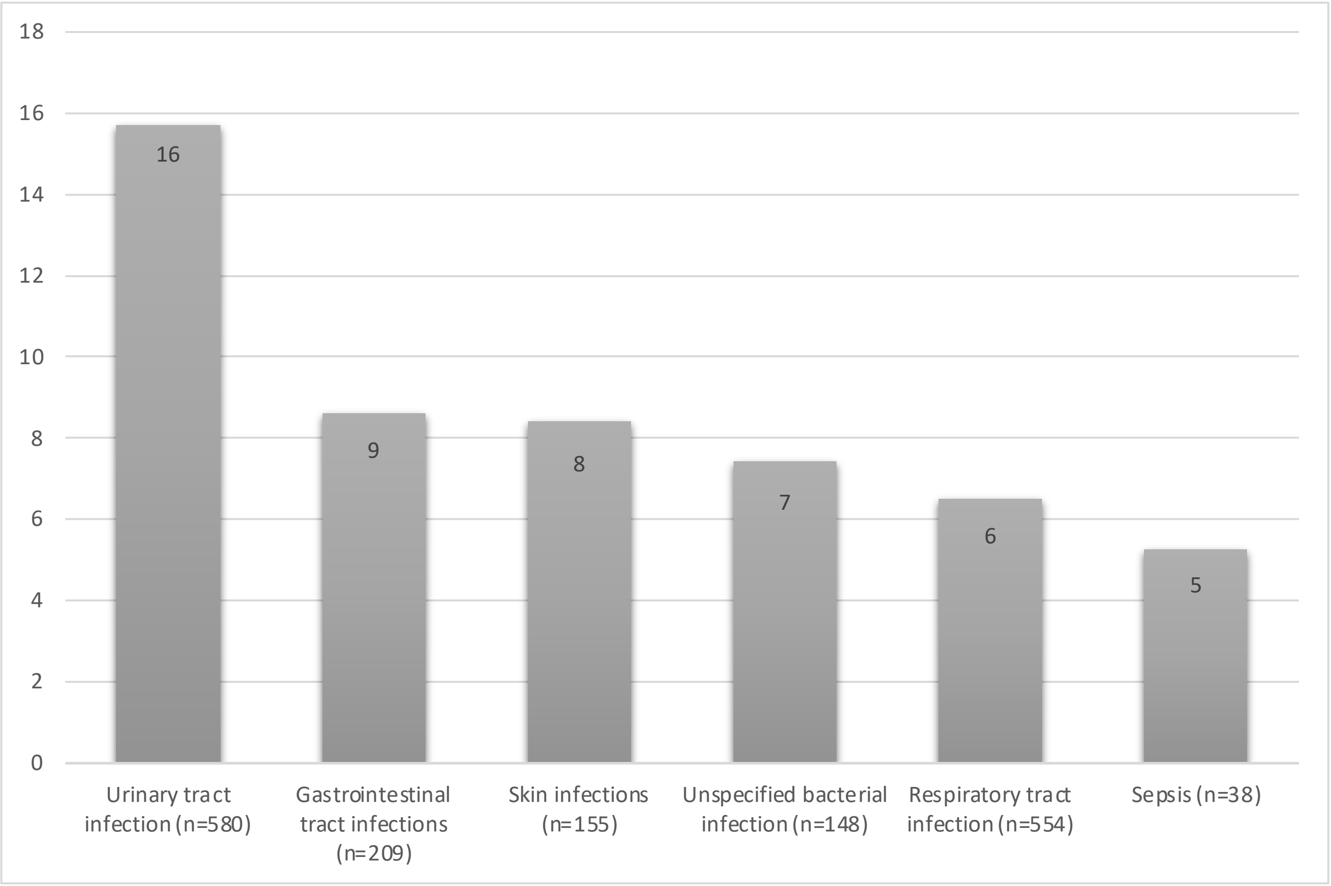
18
16
14
12
10
8
6
4
2
0
16
9
8
7
6
5
Urinary tract infection (n=580)
Gastrointestinal tract infections (n=209)
Skin infections (n=155)
Unspecified bacterial infection (n=148)
Respiratory tract infection (n=554)
Sepsis (n=38)
