# Supplementary material for: Diagnosis of urinary tract infection in the emergency department – a real infection or an alarming indication for comprehensive geriatric assessment? A retrospective follow-up study
Source: J Frailty Aging. 2026 Sep 12;15(5):100208. doi: 10.1016/j.tjfa.2026.100208 (PMC13587059; doi:10.1016/j.tjfa.2026.100208)
Supplement: Supplementary file 1 [file mmc1.zip › SUPP.docx]

Appendix I. Baseline characteristics of the participants (n = 580)

| Characteristics | n (%) |
| --- | --- |
| Age, Md (IQR), range | 84 (79.0-88.0), 75-104 |
| Age |  |
| 75–84 | 296 (51) |
| 85– | 284 (49) |
| Gender |  |
| Female | 445 (77) |
| Male | 135 (23) |
| Diabetes^a^ | 125 (22) |
| Dementia^b^ | 170 (29) |
| Charlson Comorbidity Index |  |
| ≤2 | 254 (44) |
| >3 | 326 (56) |
| Frailty^c^ |  |
| No | 251 (44) |
| Yes | 323 (56) |
| Number of visits |  |
| 1 | 518 (89) |
| ≥2 | 62 (11) |

^a^ ICD-10 codes E10-E14

^b^ ICD-10 codes F00-F03

^c^ Clinical Frailty Scale ≥5

Appendix II. Baseline differences between ED-diagnosed hospitalized cystitis cases and hospitalized pyelonephritis cases

|  | Hospitalized cystitis  (n = 152)  n (%) | Hospitalized pyelonephritis  (n = 184)  n (%) | p-value^a^ |
| --- | --- | --- | --- |
| Age, Md (IQR), range | 86.0, (81.0–91.0), 75.0–104.0 | 83.0 (79.0–88.0), 75.0–97.0 | <0.001 |
| Female | 127 (84) | 119 (65) | <0.001 |
| Charlson Comorbidity Index >3 | 104 (68) | 121 (85) | 0.108 |
| Frailty^b^ | 91 (61) | 106 (58) | 0.602 |
| Diabetes^c^ | 36 (24) | 54 (29) | 0.243 |
| Dementia^d^ | 56 (37) | 55 (30) | 0.178 |
| GFR^e^ (n = 327) |  |  | 0.252 |
| GFR >60 | 43 (29) | 63 (35) |  |
| GFR 60-45 | 47 (32) | 43 (24) |  |
| GFR <45 | 58 (39) | 73 (41) |  |

^a^Differences between the groups were tested using Chi-squared test or Fisher´s Exact test in categorical variables and with Mann-Whitney U Test for the continuous variable

^b^ Clinical Frailty Scale ≥5

^c^ ICD-10 codes E10-E14

^d^ ICD-10 codes F00-F03

^e^ Glomerular filtration rate

Md = median

IQR = interquartile range
